# Extracranial Glioblastoma Metastasis: A Neuropathological Case Report

**DOI:** 10.7759/cureus.35803

**Published:** 2023-03-05

**Authors:** George S Stoyanov, Lilyana Petkova, Bogomil Iliev, Mustafa Ali, Borislava Toncheva, Radoslav Georgiev, Tsvetan Tonchev, Yavor Enchev

**Affiliations:** 1 Pathology, Complex Oncology Center, Shumen, BGR; 2 Neurosurgery, Medical University of Varna, Varna, BGR; 3 Maxillofacial Surgery, Medical University of Varna, Varna, BGR; 4 Radiology, Medical University of Varna, Varna, BGR

**Keywords:** maxillofacial surgery, head and neck surgery, neurosurgery, recurrence, neuropathology, extracranial metastasis, glioblastoma

## Abstract

Glioblastoma (GBM) is a central nervous system (CNS) high-grade glioma with a dismal patient prognosis. Classical concepts surrounding GBM development and progression indicate its ability to produce metastasis within the CNS, one of the few primary tumors with such capabilities. While classical concepts state that no primary CNS tumor produces extracranial metastasis, there have been multiple reports of such occurrences over the previous two decades. Here, we report a case of a male in his forties who presented to our institution with complaints of progressive headache and a history of right temporal craniotomy one month prior with a histologically verified GBM performed at another institution. Neuroradiology confirmed a residual tumor in the areas of the previous craniotomy, and gross total excision confirmed the diagnosis of GBM, although based on the presence of connective tissue amidst the tumor stroma, gliosarcoma could not be ruled out. The patient initiated treatment, and his condition remained stable for four calendar years until he again presented to our institution with a rapidly growing tumor mass in the right lateral neck region. Excision of the neck mass showed histopathological features of a tumor comprised of atypical cells with pronounced polymorphism, some with spindle cell morphology and a tendency for fascicular growth and focal palisade necrosis. Immunohistochemistry with a broad set of markers disproved epithelial, mesenchymal, melanocytic, and lymphoid genesis, with some markers of glial genesis present; hence, metastatic GBM was established. The patient reinitiated treatment and is currently stable. The steadily increasing amount of similar reported cases, together with the steady, albeit small, increase in GBM patient survival and improvement of neurooncological healthcare distribution and follow-up, challenge the classical concepts of GBM and other primary CNS tumors being unable to produce metastasis and swaying this perception towards the biological capabilities of these tumors to produce metastasis, while such rarely develop due to the short patient survival.

## Introduction

Glioblastoma (GBM), a World Health Organisation (WHO) central nervous system (CNS) grade 4 astrocytoma, is defined by lack of isocitrate dehydrogenase (IDH) mutation, +7/-10 chromosome aberration, telomerase reverse transcriptase (TERT) promoter mutation, epidermal growth factor receptor (EGFR) gene amplification, microvascular proliferation, or tumor necrosis, and is a malignant tumor with dismal prognosis [1]. Classically, GBM is the only CNS tumor that can produce metastasis; however, that refers to ventricular/cerebrospinal fluid dissemination and not true metastasis [2].

Over the past couple of decades, with significant research and leaps in our understanding of the molecular biology of glial neoplasms, there has been a small increase in the overall survival of GBM patients from around 12 months to more than 16 months in most developed countries. This relatively small increase, together with better access to neurosurgery, has significantly increased the remaining life quality in GBM patients; however, it has also led to multiple reports contradicting the classical dogma that CNS tumors do not produce systemic metastasis, indicating its outdated nature, albeit with a low incidence rate compared to tumor incidence itself.

Initial reports were of shunt metastasis to the peritoneum and pleura; however, in recent years, soft tissue, bone, and lymph node metastasis have also been reported in significant numbers [3,4]. These further raise the question of whether GBM is incapable of producing metastasis, based on its native cell biology; do treatment-induced and associated mutations allow for their development, or are GBM, like most other tumors, a tumor with pronoun metastatic potential, but the patients, due to its exceedingly fast growth (GBM double in size in 15-30 days), do not survive long enough for them to develop and clinically manifest?

## Case presentation

A 41-year-old male patient presented to our healthcare institution with complaints of progressive headache and a history of right temporal craniotomy one month prior in another healthcare institution. Indications for the previous neurosurgical intervention were presenting symptoms of headache, nausea, and vomiting, progressing over the past couple of weeks, with an outpatient computer tomography showing a temporal tumor formation. A craniotomy with subtotal resection was performed with a histologically verified GBM.

**Figure 1:**
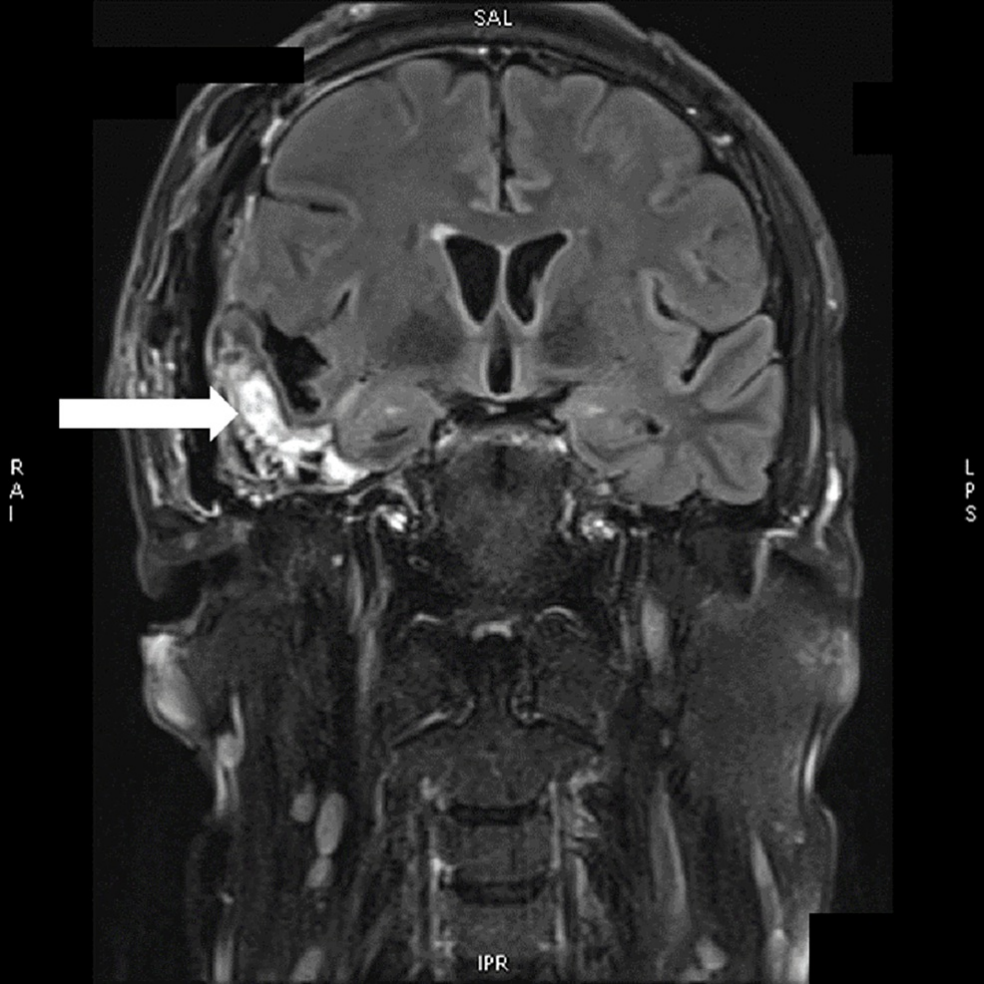
COR T2 TIRM postcontrast MRI of the brain showing postoperative changes in the right temporal lobe with residual tumor tissue (arrow) COR T2 TIRM: coronal, transverse relaxation time turbo inversion recovery magnitude; MRI: magnetic resonance imaging

**Figure 2:**
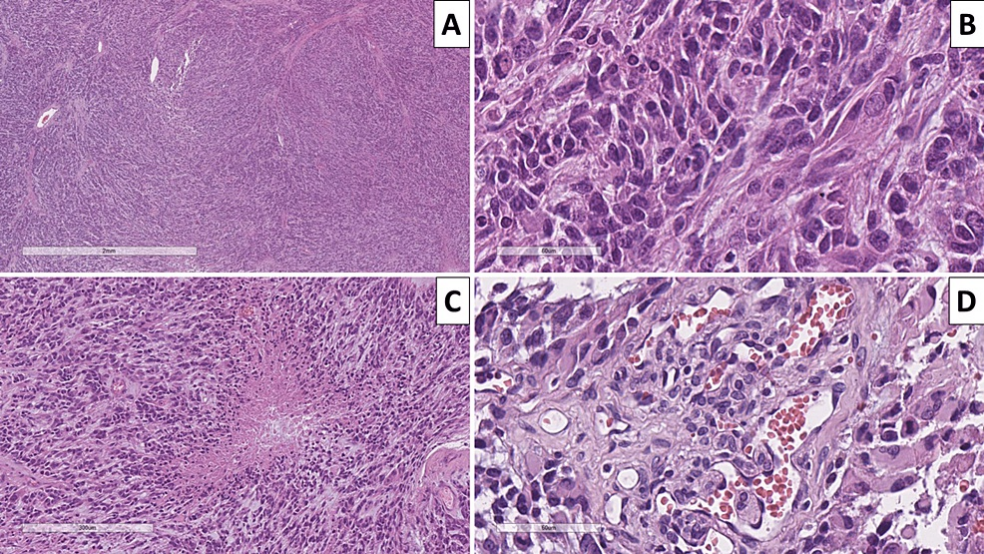
Histopathology of the intracranial lesion A: hypercellular lesion, hematoxylin and eosin, original magnification x40; B: sarcomatoid areas, hematoxylin and eosin, original magnification x400; C: pseudopalysadic necrosis, hematoxylin and eosin, original magnification x200; D: glomeruloid vascular proliferation, hematoxylin and eosin, original magnification x400

**Figure 3:**
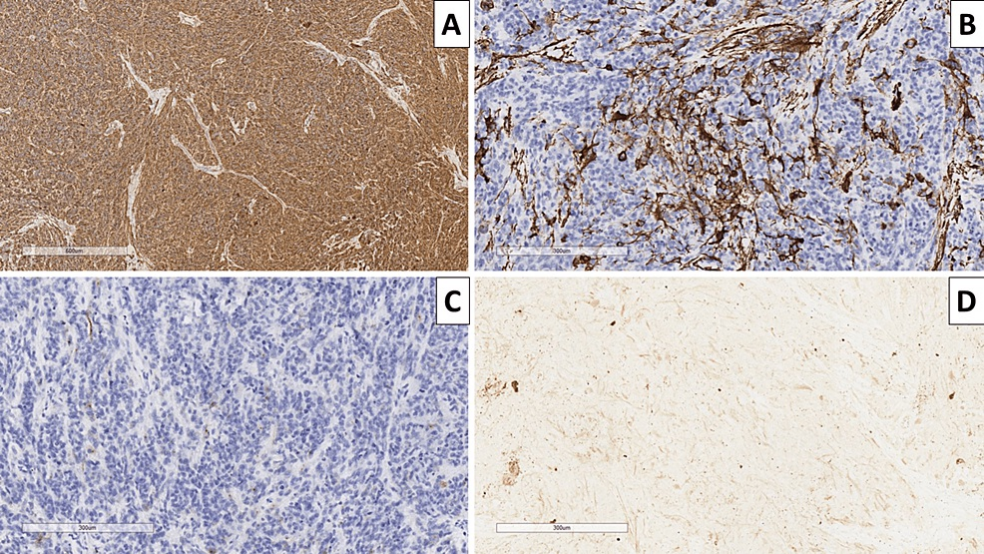
Immunophenotype of the tumor A: glial fibrillary acidic protein, original magnification x100; B: smooth muscle actin, original magnification x100; C: epithelial membrane antigen, original magnification x100; D: pan-cytokeratin, original magnification x100.

Upon presentation to our institution, neuroradiology showed a residual tumor in the area of the previous neurosurgical intervention (Figure 1). The patient was scheduled for a neurosurgical intervention for the removal of the residual tumor, with neuropathology confirming the diagnosis of GBM; however, due to areas of connective tissue proliferation, the diagnosis of gliosarcoma, a rare subtype of GBM, was also suggested (Figures 2-3). In this respect, neuropathology could not be definitive due to the previous neurosurgical intervention and the short time span between the two.

After surgery, the patient was regularly followed up in an outpatient setting and received six cycles of temozolomide, which were well tolerated. The patient further underwent volumetric modulated arc therapy to a total of 60 Grays (Gy), split up into 2 Gy doses.

For a period of four calendar years, the patient remained neurologically stable without complaints and was regular to follow-ups, with neuroradiology showing no disease progression or recurrence.

**Figure 4:**
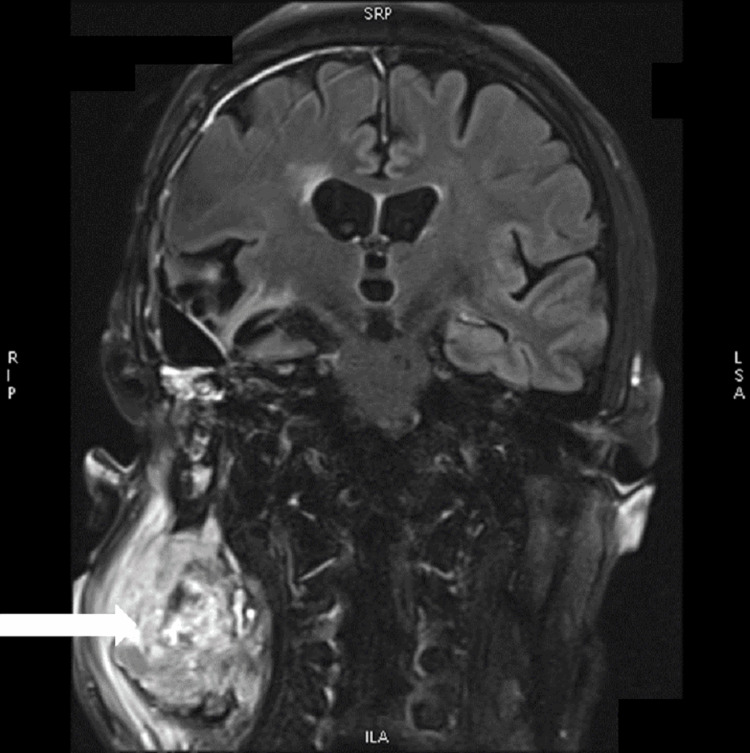
COR T2 TIRM postcontrast MRI of the brain showing the persistent postoperative changes in the right temporal lobe and a big heterogenous contrast-enhancing tumor in the neck on the right side (arrow) COR T2 TIRM: coronal transverse relaxation time turbo inversion recovery magnitude; MRI: magnetic resonance imaging

About a month prior to the current presentation, the patient noticed a rapidly growing painless swelling in the right lateral neck region. Due to the deterioration of the patient's general condition, he was admitted to the department of maxillofacial surgery for the removal of the formation, which in the outpatient setting was determined to be enlarged lymph nodes. The clinical exam showed a firm area posterior to the sternocleidomastoid muscle, lobulated, painless, and fixed to the surrounding tissue. Imaging modalities did not show direct invasion into the surrounding tissues (Figure 4). Excision was performed under general anesthesia with total extirpation of the formation, which was found to be easily bleeding. The postoperative period was uneventful.

**Figure 5:**
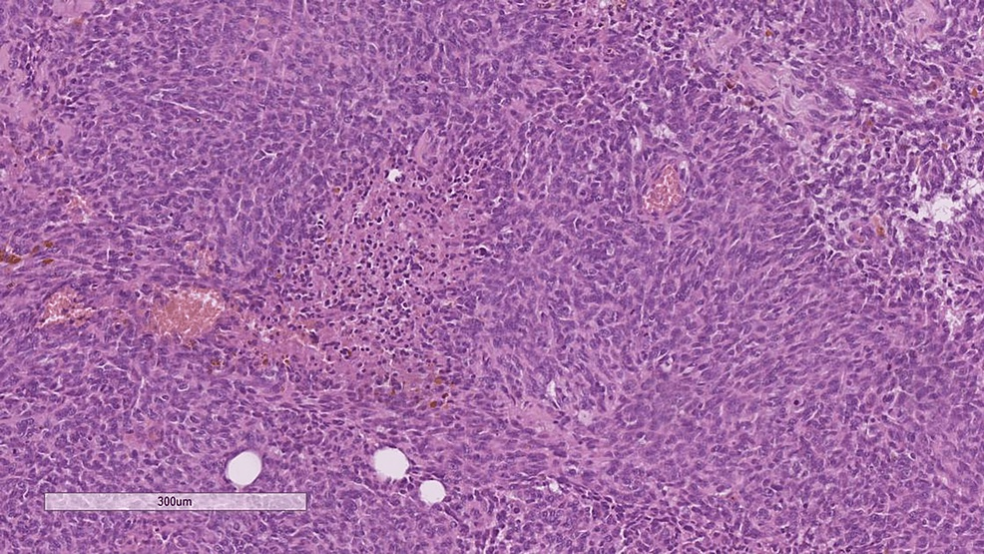
Histopathology of the neck mass, showing pseudopalisadic necrosis and a poorly differentiated cellular population, hematoxylin and eosin stain, original magnification x100

Specimens sent for histopathology revealed fibrous and adipose tissue with tumor infiltration from atypical cells with pronounced polymorphism, some with spindle cells morphology and a tendency for fascicular growth and focal palisades necrosis, groups of pigmented macrophages in the tumor stroma around focal hemorrhages, and stromal myxoid change (Figure 5).

**Figure 6:**
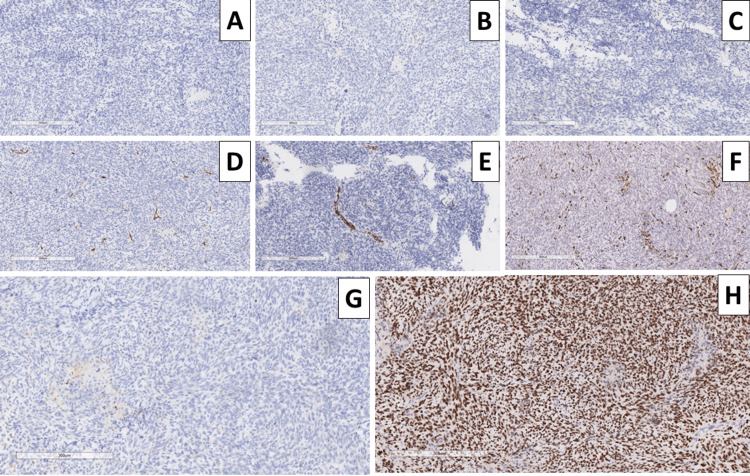
Immunophenotype of the neck mass A: glial fibrillary acidic protein, original magnification x100; B: S100 protein, original magnification x100; C: epithelial membrane antigen, original magnification x100; D: CD34, original magnification x100; E: smooth muscle actin, original magnification x100; F: CD68, original magnification x100; G: pan-cytokeratin, original magnification x100; H: p53 protein, original magnification x100. CD: cluster of differentiation

Due to the patient already having a histologically proven malignant tumor, GBM, and despite the rarity of reported extracranial GBM metastasis and the broad differential diagnosis of the tumor histomorphology, a comprehensive set of immunohistochemical markers were used. Pigmented cells within the tumor were cluster of differentiation (CD) 68 positive, with the tumor cells being S100 negative, excluding malignant melanoma; smooth muscle actin (SMA) was negative, excluding leiomyosarcoma; pan-cytokeratin (CK AE1/AE3) and epithelial membrane antigen (EMA) were negative excluding a spindle cell epithelial malignancy; CD34 was positive in vascular endothelial cells and focally within the tumor cells; while glial fibrillary acidic protein (GFAP) was negative, tumor protein P53 was diffusely positive (Figure 6).

Based on the morphology and immunophenotype of the tumor, as well as the patient's clinical history of ipsilateral temporal GBM, the tumor mass was interpreted as a metastatic GFAP-negative GBM. The oncologic committee reevaluated the patient, and GBM treatment was reinitiated. Several months after the operation, the patient is stable, without local or CNS recurrence and progression of GBM.

## Discussion

As already mentioned, metastatic GBM is exceedingly rare, despite a steady increase in reported cases. As seen in our case of a long-GBM survivor, there may be an association between the prolonged survival of these patients in recent decades and the development of extracranial metastasis; as historically, patients may not have survived long enough for their development and clinical manifestation, or these metastases may have been interpreted as separate tumors, such as malignant peripheral nerve sheath tumor, leiomyosarcoma, fibrosarcoma, etc. Table 1 summarizes the findings in other similar cases published in the head and neck area.

**Table 1:** Summary of the clinical and morphological profile of published cases with metastatic glioblastoma IDH: isocitrate dehydrogenase; GFAP: glial fibrillary acidic protein; LN: lymph node; MM: masseter muscle; p53: transformation-related protein 53

Author	Gender	Age	Lobar location	Immunophenotype of the primary tumor	Metastatic site	The time interval from operation/biopsy to metastasis (months)	Immunophenotype of the metastasis	Postmetastatic survival (months)
Waite et al., 1999 [5]	Male	40	Frontal	GFAP(+),	LN	24	GFAP(+/-),	2
Taha et al., 2005 [6]	Male	33	Frontal	GFAP(+),	LN	5.5	GFAP(-)	3
Ogungbo et al., 2005 [7]	Female	49	Occipital	unknown	LN	5	unknown	9
Kraft et al., 2008 [8]	Male	58	Temporal	unknown	Orbit, lung, pleura, mediastinal LN, Thoracic spine, femur, liver, left auricle of the heart	15	unknown	2
Taskapilioglu et al., 2013 [9]	Female	30	Frontal	GFAP(+), p53(+),	LN, bones	10	unknown	6
Romero-Rojas et al., 2013 [10]	Male	26	Frontal	GFAP(+)	LN, bones	6	GFAP(+),	18
Swinnen et al., 2019 [11]	Female	56	Temporal	GFAP(+)	LN, Lungs	3.5	GFAP(+)	11
Alhoulaiby et al., 2020 [12]	Male	53	Temporoparietal	unknown	LN, MM,	6	GFAP(+),	4
Baskurt et al., 2022 [13]	Male	42	Temporal	GFAP(+)	Cervical LN	15	GFAP(+/-)	24 (still alive)

Another rare feature of our case is that despite the patient's clinical history, the ipsilateral neck mass, and the classical histomorphological findings, the tumor is GFAP negative. While GFAP is considered the most specific marker for astroglial differentiation, it is worth noting that studies have demonstrated GFAP negativity in tumors, some associating it with further anaplasticity [14,15]. This loss of GFAP is not only due to the loss of antibody reaction, which may be specific to the clone of the antibody used, but due to the actual loss of cytoplasmic GFAP, as a result of decreased protein production [16].

As seen by the influx of published articles on the topic, these conditions are either a new aspect of glioma pathology or are just starting to be reconsidered and not misinterpreted [17-19]. Cervical masses, however, seem to be quite a common occurrence in the context of metastatic disease, as seen by the case reports published by Sun et al., Waite et al., Taha et al., and Baskurt et al., with almost all of the reported patients being males in their forties [5-6,17].

Additionally, classically GBM can arise in extracranial sites based on precursor teratomas [20]. In our case, however, as well as the cases cited so far and based on the location of teratomas, this cannot be taken into account due to the presence of a previous histologically verified GBM.

An interesting aspect of CNS morphology, with potential implications for neurooncology as a whole over the past several years, has been the depiction of functional glial lymphatic-like systems, also referred to as the glymphatic system [21]. Unlike in other systems where there is a dedicated lymphatic system, within the CNS, debris and metabolic clearance is achieved through a semicontinuous system consisting of the cerebrospinal fluid, periarterial spaces (so-called Virchow-Robin spaces), interstitial fluid, perivenous spaces, and the extracranial lymphatics around the large veins (cervical lymphatic systems). This semicontinuous system allows for a continuous exchange of fluids, with the cerebrospinal fluid being pressure pumped into the periarterial Virchow-Robin spaces, which disappear shortly before the capillary branches. From the discontinued Virchow-Robin spaces, the cerebrospinal fluid is pumped into the interstitium and mixes together with the interstitial fluid, which then drains into Virchow-Robin-like perivenous spaces, flowing alongside the dural sinuses and then draining into the cervical lymphatics surrounding the large veins at the base of the skull [21]. Such a continuous flow of extracellular and extravascular fluid ensures constant extracellular clearance, which is vital for the CNS's metabolism and homeostasis and has many implications for the development and progression of neurodegenerative diseases [21]. Based both on the depicted morphology and function of the glymphatic, as well as the preference of GBM to produce metastasis in the cervical region, the combination of these should serve as a future perspective for research towards the potential of the glymphatic system to also serve as a highway for tumor dissemination, not only outside the CNS but also within and towards it.

## Conclusions

GBM is a malignant CNS tumor with a historically dismal prognosis, which classically does not provide extracranial metastasis. In recent decades, however, with the steady increase in survival of GBM patients, such cases have been published with increased frequency. The reasons for this can be that, based on the aggressive biological behavior of the tumor, patients have so far not survived long enough for such metastases to develop and manifest clinically or have been interpreted as synchronous histogenetically different tumors.

As seen in our case, cases of extracranial tumors in patients with GBM require a careful interpretation of histological, immunohistochemical, and clinical data, as despite being in line with classical morphology, the tumor in our case was GFAP negative.
